# Micromachined needle-like calorimetric flow sensor for sap flux density measurement in the xylem of plants

**DOI:** 10.1038/s41598-024-65046-9

**Published:** 2024-06-27

**Authors:** Gigyu Kim, Junghoon Lee

**Affiliations:** 1 Department of Mechanical Engineering, College of Engineering, Seoul National University, Seoul, 08826 Republic of Korea; 2 Telofarm, Inc., Seoul, 06766 Republic of Korea

**Keywords:** Plant sciences, Engineering

## Abstract

Miniaturized silicon thermal probes for plant’s sap flow measurement, or micro sap flow sensors, have advantages in minimum invasiveness, low power consumption, and fast responses. Practical applications in sap flow measurement has been demonstrated with the single-probe silicon micro sensors. However, the sensors could not detect flow directions and require estimating zero sap flow output that leads to significant source of uncertainty. Furthermore, silicon-needles would break easily during the insertion into plants. We present the first three-element micro thermal sap flow sensor packaged on a durable printed circuit board needle that can measure bidirectional flows with improved dynamics and precision. The performance of the newly designed calorimetric flow sensor was confirmed through precision calibration and field test on tomato stems. A calibration curve for a tomato stem was obtained with a sensitivity of 0.299 K/(µL mm^−2^ s^−1^) under the maximum temperature increase of 4.61 K. Results from the field test for one month revealed a correlation between the measured sap flux density and related conditions such as solar radiation, vapor pressure deficit, sunshade and irrigation. The developed sensor will contribute to practical long-term sap flow monitoring for small and delicate plants with minimal physical invasion.

## Introduction

Water movement in plants is indispensable for obtaining physiological information that may be required in a variety of applications. Transpiration-driven sap flow in the xylem plays a key role in distributing nutrients and hormones, supplying water for photosynthesis, cooling, and maintaining turgor pressure and water balance. The precise measurement of this flow rate, which reflects the direct response of plants to biological or abiotic variable changes, is a prerequisite for investigating its underlying physiological mechanisms, which in turn leads to practical applications such as water resource management^1^, ecosystem modeling^2^, urban forest planning^3,4^, and phytoremediation^5^. In agricultural science, sap flow monitoring facilitates not only water use optimization^6,7^ and auto-irrigation scheduling^8,9^ but also disease detection^10–12^.

Since Huber^13^ estimated the sap flow rate by measuring the heat-pulse travel time, most of sap flow sensors are based on the principle of thermal flow meters. These sensors could be categorized into three types: hot-wire, calorimetric, and time-of-flight. The heat dissipation^14^ (HD) method adopts a hot-wire configuration and is the most widely used setup owing to its simplicity and low cost. The setup consists of a heater-thermometer probe and a reference probe to cancel out the ambient temperature drift. For example, two probes (2 mm in diameter and 20 mm in length) are inserted radially into the sapwood with an axial distance of 10 cm. The temperature difference between the heater and reference probe, which are both subjected to sap flow that convectively cools the heater, is measured under continuous Joule heating of the downstream heater and converted into a measurement of the sap flux density (SFD, µL mm^−2^ s^−1^) based on the experimentally obtained regression curve. This calibration curve is in the form of a power-law model which can be derived from King’s law^15^ for hot-wire anemometry.

The heat field deformation^16^ (HFD) method, which is synonymous with calorimetric sensing, correlates the thermal profile around the constant heat source to the SFD. This method measures the temperature difference between two thermocouple probes arranged equidistantly upstream and downstream from a centered heater at an interval of, e.g., 1.5 cm. Another probe is installed 0.5 cm in the tangential direction away from the heater to measure the temperature difference to the upstream thermocouple, which expands the measurement range of the HFD method to higher SFD ranges. A purely empirical formula was suggested to extract the SFD. This formula is a linear function of the ratio of the recorded temperature differences between the probes.

The heat-pulse method, which is grouped into a time-of-flight technique, relates temporal changes in the temperature of the sensing probes after a heat pulse is applied to the sap flux, whereas the other methods are based on the spatial temperature difference between the probes during constant heating. Diverse heat-pulse methods have been developed based on the analytical solution of the temperature field of a heat pulse in a moving medium presented by Marshall^17^. For instance, the compensation heat-pulse velocity^18^ and Tmax^19^ methods compute the SFD using the time at which the temperatures of the two probes, positioned unequally up and downstream of the heater, equalize, and the time taken for the downstream probe to reach its maximum temperature, respectively. The heat ratio^20^ (HR) method determines the SFD through the average ratio of the temperature rise in equidistant upstream and downstream probes between 60 and 100 s after the application of a heat pulse. These heat pulse-based estimations of SFD are rooted in a well-defined analytical background that makes empirical calibration unnecessary. However, depending on the estimation method, the measurement range and frequency of heat-pulse technique are limited^21^.

The invasive nature of the sap flow sensors described above poses a critical issue in practical applications for real plants. First, installing a probe in the sapwood leads to a mechanical wound caused by drilling a hole or inserting the probes. Tyloses, which are balloon-like distensions of xylem parenchyma cells that form after this wound, partially or fully occlude the lumen of the xylem vessels around the probes^22^. The occlusion and obstruction caused by the sensor structure lead to a decrease in hydraulic conductivity of the xylem near the sensor, thus resulting in an underestimation of the SFD and sensitivity degradation of the sensor. This error and performance degradation are proportional to the extent of the wound^23^; however, the wound reaction of plants is challenging to predict because it depends on various factors, such as the species, growth rate, season, climatic conditions and sensor location^24–27^.

One strategy for minimizing wound-induced errors is to reduce the size of the sensor and the number of probes employed in the measurement system^28^. Diminishing the physical stress on plants through sensor installation also expands the applicable range of conventional sap flow sensors to small and delicate plants. Recently, microscale sap flow sensors fabricated with micro-electromechanical systems (MEMS) technology have been proposed. Baek et al.^29^ measured the sap flow in tomato vines using a modified HD method in which a needle-like silicon probe acts as heater-thermometer and reference probes by capturing unheated and heated temperatures under periodic heating. Hara et al.^30^ and Yano et al.^31^ relied on very small dimensions of silicon probes intended to monitor sap flow in plant shoots, although they only demonstrated laboratory calibration for water flow in a circular tube.

Despite the benefits of miniaturization, such as low power consumption, fast response, high sensitivity, and minimal invasion, micromachined sap flow sensors have issues in practical applications. The silicon-needles would break easily during the insertion into plants^29^. Furthermore, the “single-probe” modified HD method is incapable of detecting flow direction and canceling out ambient temperature drift during the heating period. Also, the HD method requires determining the maximum temperature difference at zero sap flow that leads to a significant source of uncertainty^32^.

We present the first single-probe three-element micro thermal sap flow sensor based on a calorimetric sensing system that shows improved dynamics and precision in low flow rates compared to hot-wire sensors^33^. The key feature of the micromachined calorimetric flow sensor is minimized thermal conduction between the heater and thermometers, thereby enhancing sensitivity. This thermal isolation is typically achieved by placing the sensing elements on a freely suspended thin beam in the flow channel^34^, on a back-etched membrane under which the fluid flows^35^ or on a substrate with low thermal conductivity^36,37^. In our approach, silicon substrates laid out on a laser-cut printed circuit board (PCB) are completely divided into three separate silicon bodies with encapsulation of low thermal conductive underfill epoxy. The precision integration of three sensing components onto a single PCB probe (880 µm wide, 300 µm thick) gives rise to microscale, but robust sensing system suitable for unbreakable installation and long-term field measurement of sap flow in plants, and eliminates the error resulting from misalignment^38^ that can occur with the conventional multi-probe systems. Moreover, our three-element configuration enables bidirectional measurements and insensitive output to ambient temperature fluctuations.

## Design and fabrication

### Design and simulation

**Figure 1:**
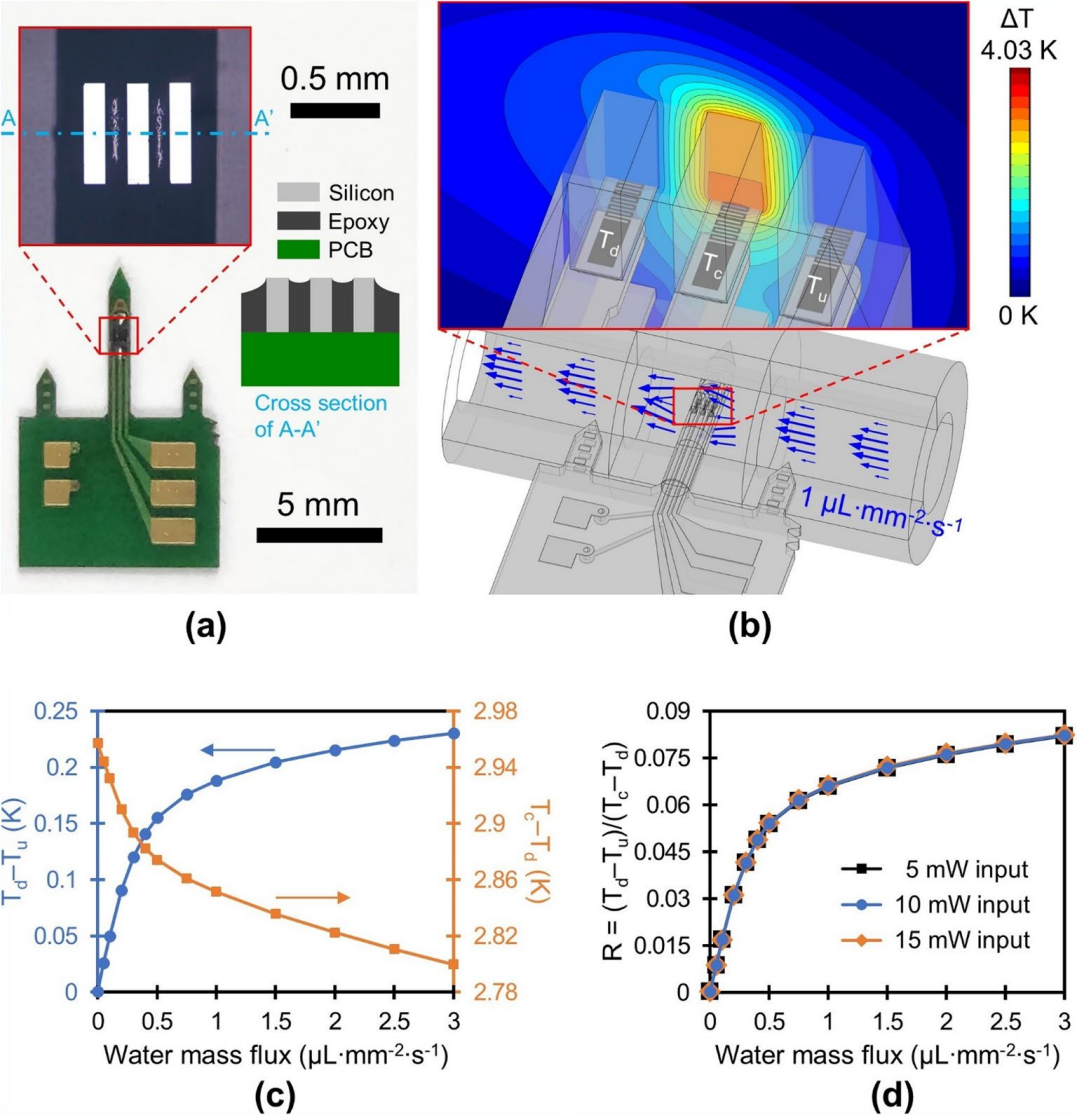
Design and simulation results of the developed sensor. (**a**) An image of the packaged sensor along with a microscopic view of the sensing elements and its cross-sectional view (microstructures and copper traces are omitted). Grey lines between sensing elements in the microscopic image are reflected light source on the epoxy surface. (**b**) Simulation model of the sensor, illustrating temperature distribution in a central plane through the tube at 1 µL mm^−2^ s^−1^ flow and 10 mW power. (**c**) Two differential temperatures at varying mass fluxes and (**d**) their ratio for 5, 10 and 15 mW of input power (COMSOL Multiphysics 5.6, https://www.comsol.com).

A photograph of the packaged sensor is shown in Fig. 1a. The calorimetric sap flow sensor consists of a laser-cut printed circuit board (PCB) and micromachined sensing components. The shaped PCB includes a needle-like probe, double-sided wiring board, and two subsidiary needles that assist clinging to the plant stem. On the PCB probe, which is 880 µm wide, 300 µm thick and 6 mm long, three silicon bodies are mounted and lined up parallel to the flow direction (displayed in the inset of Fig. 1a). These cuboidal silicon islands that are 550 µm in length, 120 µm in width, and 300 µm in thickness, are separated by an underfill epoxy with an interspace of 120 µm. Meander-shaped thin film titanium resistive heater/thermometer structures, 290 Ω in electrical resistance, are buried underneath each silicon substrate (Fig. 1b). The silicon substrates, encapsulated with underfill epoxy while leaving the top surface exposed, act as thermal conduction elements (130 W m^−1^ K^−1^) to bridge the temperature of the fluid above the sensor to that of the titanium thin film. An underfill epoxy without filler material is selected for its low thermal conductivity (0.2 W m^−1^ K^−1^) to reduce thermal conduction between silicon substrates. Three contact pads on the right side of wiring board shown in Fig. 1a are connected to titanium resistive structures at three different locations: upstream (T_u_), downstream (T_d_), and center (T_c_) (Fig. 1b). Remaining two contact pads on the left side of the board are ground connections (one for both up and downstream resistive thermometers and the other for centered resistive heater/thermometer), which are routed on the back side of the board. 1.7 V is applied across the center resistive heater/thermometer, resulting in a Joule heating of approximately 10 mW. The rise in resistance of the three resistive thermometers after 5 s of heating is captured using a Wheatstone bridge and converted into temperature elevation through consideration of temperature coefficient of resistance of the titanium. Across both up and down thermometers 0.02 V is applied in order to minimize the error resulting from self-heating.

The water mass flux is estimated by characterizing the temperature profile near the sensor upon forced convection. As the flow velocity increases, the symmetrical temperature distribution around the heat source at zero flow is gradually distorted. We ascertained the feasibility of our new design through 3D simulations. The temperature field surrounding the sensor was solved using conjugate heat transfer model in COMSOL Multiphysics 5.6 under the assumptions: fully developed laminar flow, non-slip boundary condition, and thermally thin layer. We modeled water flowing through a 3 mm inner diameter silicone tube to represent the sap flowing in the xylem. Figure 1b displays the temperature distribution solved for the silicone tube, water stream, MEMS structure, electrical leads, and PCB under a water mass flux of 1 µL mm^−2^ s^−1^. The temperature difference between the top and bottom sides of the silicon body and among the silicon bodies indicates that the silicon body functions well as a thermal conduction element and the underfill epoxy provides effective insulation.

The simulation results displayed in Fig. 1c illustrate the influence of various water mass fluxes on the thermal profile. The temperature difference between the upstream and downstream resistive structures, T_d _– T_u_, increases linearly at low water mass fluxes, which is distinctive response of the calorimetric sensor at low flow rates that can be derived through perturbation approaches^39,40^. The sensitivity of the sensor gradually diminishes when exposed to higher flow rates, as a consequence of a decline in heater temperature^34^ and the development of a thermal boundary layer^41^, or specifically the detachment from the channel wall beginning upstream. Xu et al.^41^ proposed Peclet number ($\text{Pe}$) $\approx 1$ criterion where non-linear response of a calorimetric sensor emerges based on thermal boundary layer analysis. Our design shows non-linear response around $\text{Pe}=0.82$ (0.5 µL mm^−2^ s^−1^). The temperature difference between the heater and downstream thermometer, T_c _– T_d_, monotonically decreases as the flow velocity increases, and declines by only 5.5% at a water mass flux of 3 µL mm^−2^ s^−1^ compared to zero flow. We correlate the water mass flux with the ratio of the two differential temperatures (R) as

1 $$R=\frac{{T}_{d}-{T}_{u}}{{T}_{c}-{T}_{d}},$$

where the divisor that remains relatively constant upon variation in the flow velocity as opposed to the numerator, contributes negligibly to the sensitivity of the sensor. The fractional form of the two differential temperatures (R) normalizes the influence of the applied power on the sensor response in view of the fact that temperature gradient around the heat source is directly related to the heating power^34^. As a result, the simulation demonstrates that the sensor response is independent of the input power (Fig. 1d). This independence enables a simple constant-voltage operating mode that does not require any complex feedback components. Also, the difference between two temperatures measured in close proximity (the divisor and numerator) ensures output insensitive to ambient temperature fluctuations^35^.

### Fabrication and packaging

**Figure 2:**
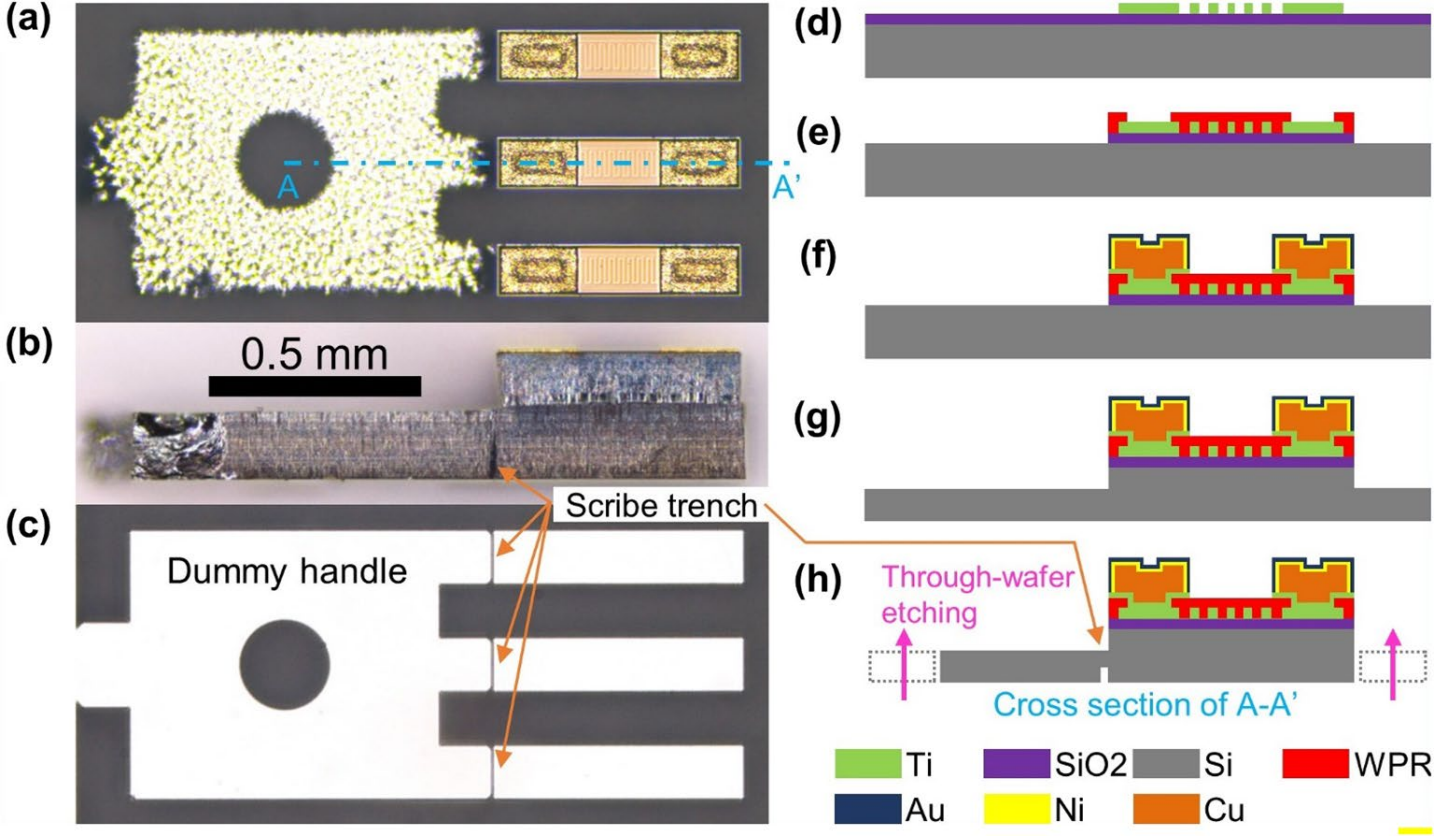
Fabrication process of the sensing chip. A microscopic image of the fabricated chip with views from (**a**) top, (**b**) side and (**c**) bottom. The orange arrows point to scribe trenches along the [110] direction of the wafer, for dummy separation in the packaging step. Schematic representation of the process: (**d**) resistive structure and (**e**) insulation layer patterning, (**f**) mini bump plating, and deep silicon etching on (**g**) front side and (**h**) back side.

Microscopic images of the micromachined silicon chip are displayed in Fig. 2a–c. Figure 2d–h illustrates the fabrication process of the silicon chip. The overall die size is approximately 1.5 mm × 0.7 mm including an auxiliary structure called dummy handle, which is eliminated in the packaging step (Fig. 2c). P-type silicon, 4-inch, and (100)-oriented wafer was used as a substrate. The wafer was double-side polished down to a thickness of 300 µm to reduce the thermal conduction area among sensing elements and obstructive protrusion during installation. In the first step of the fabrication process, a layer of silicon dioxide (SiO_2_) insulation with a thickness of 1 µm was deposited on top of the bare substrate using high density plasma-chemical vapor deposition equipment. A 4000 Å thick titanium film was sputtered, and 5 µm broad meander-shaped titanium resistive structures were defined after photolithography and etching with an inductively coupled plasma-reactive ion etching process (Fig. 2a,d). After the buffered oxide etching of the SiO_2_ layer in preparation for deep silicon etching, a polyimide-based photoresist (WPR-1021, JSR) was patterned as an insulation layer, which leaves pads for electrical connections (Fig. 2e). A 10 µm thick plating templet was structured with a positive photoresist (AZ4620, Microchemicals) subsequent to the deposition of a 150 Å titanium adhesion layer and a 1000 Å copper seed layer through sputtering. Copper pillars of height approximately 5 µm were electroplated on the exposed pads as mini bumps for flip chip bonding. A 1-µm thick nickel and a 0.3-µm thick gold layer were electroless plated onto the copper pillars to serve as a diffusion barrier and surface finish, respectively (Fig. 2f).

Finally, chip fabrication was completed by deep reactive ion etching (SLR-770-10R-B, Plasma Therm) of the silicon substrate on both sides. 120 µm deep silicon etching on the front side defined the boundaries of the upper side of the rectangular silicon structures (Fig. 2a,g), and subsequent 180 µm through-wafer etching on the back side resulted in the floating silicon bodies affixed to the dummy handle (Fig. 2c,h). This handle transfers the arrangement of the silicon substrates onto the PCB exactly as it stands. At the joint of the handle and the actual chips, a scribe line of 80 µm deep trenches^42^ along the [110] direction is included in addition to the backside etching by controlling the ratio of mask opening (1:50 in our case) for the scribe trench and the main structure (Fig. 2b,c,h). The fabricated chip was mounted upside down on the PCB and aligned with the matching pads to which the solder paste was applied prior to picking and placing. Following solder reflow, applying gentle pressure on the dummy handle caused cracks that naturally propagate along the scribe line of the [110] direction^43^, thereby eliminating the handle from the silicon chips. An underfill epoxy (WE-1007, Won Chemical) dispensed along the sides of the remaining three chips filled the gaps between the silicon bodies through capillary action. A newly designed calorimetric sap flow sensor was obtained through a laser cutting of the PCB. With a simple modification of the PCB design, an array of sensing silicon chips can be mounted on the PCB needle to discern the radial profile of the sap flow in large woody plants. However, this study focused on monitoring the sap flow in small plants.

## Results and discussion

### Experimental calibration

**Figure 3:**
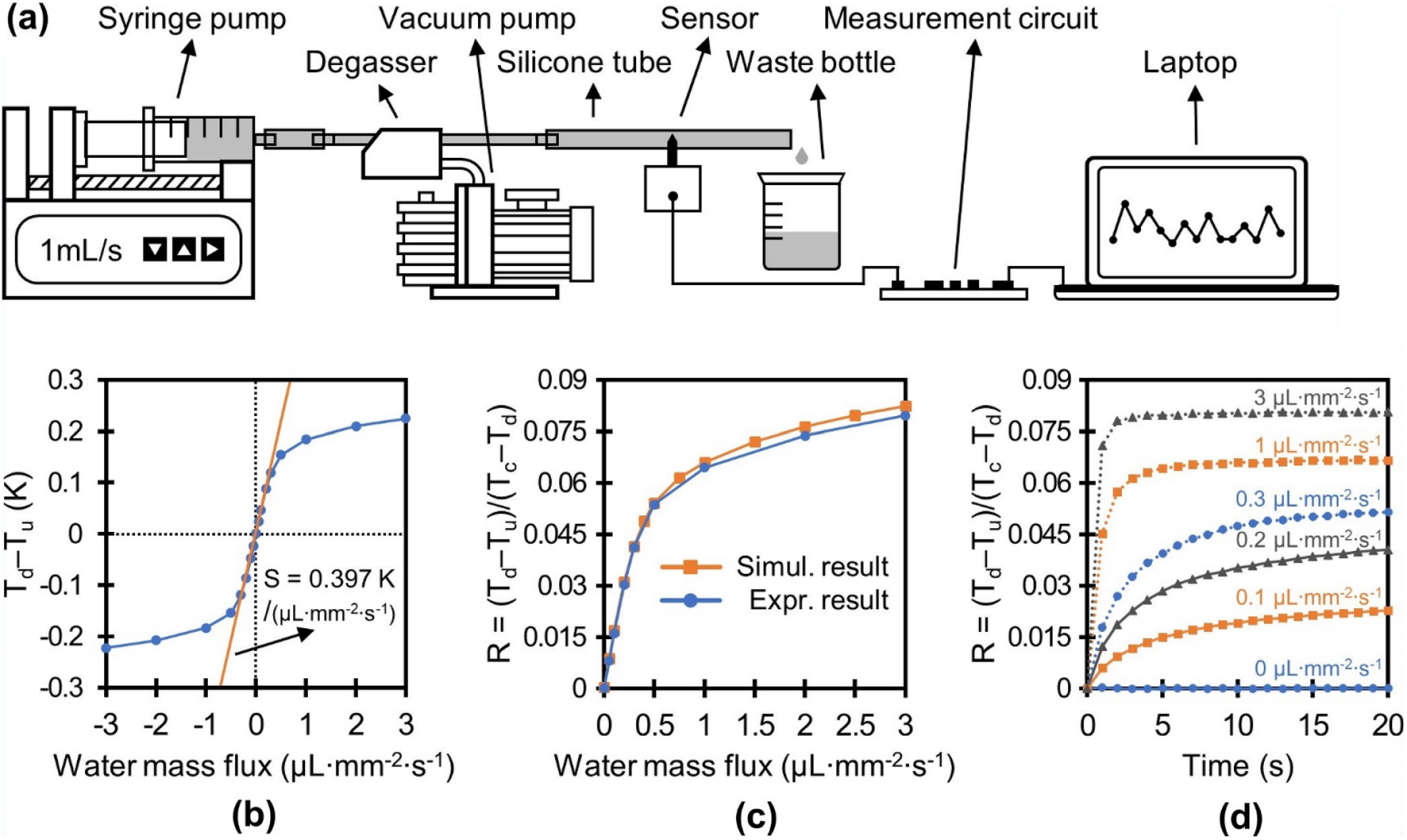
Experimental setup for the tube test and the results. (**a**) Schematic representation of the calibration setup. (**b**) Temperature difference at up and downstream thermometer at diverse mass fluxes. (**c**) Comparison of the sensor response between the results from the experiment and the simulation. (**d**) Time response of the sensor in the tube test.

The feasibility of the new calorimetric flow sensor design was confirmed through experimental calibration. First, we obtained the analytical sensitivity by correlating the water flow in a silicone tube to the measured signal. The detailed calibration setup is illustrated in Fig. 3a. The needle-like probe of the packaged sensor was inserted radially into a silicone tube 3 mm in diameter, and the central line crossing the three sensing elements was aligned with the central axis of the tube, which replicated the configuration of the simulation model. Degassed and deionized water flowed through the tube and the mass flux was regulated using a precision syringe pump (Legato 110, KD Scientific). The calibration was carried out with a mass flux of up to 3 µL mm^−2^ s^−1^, or 3 mm/s of average flow velocity, which was 10 times higher than the maximum SFD that occurs naturally in plants^16^. The temperature changes in the sensing elements were captured after a 5 s heating period at a power of 10 mW. The heater was then turned off and cooled for 55 s to complete a single measurement cycle. The sensor was connected to a custom measurement circuit and 10 consecutive measurement results for each mass flux were transmitted to a personal computer thorough asynchronous serial communication.

Figure 3b displays the relationship between the average differential temperature (T_d _– T_u_) of the 10 measurements and the water mass flux. The sensor responded almost linearly to the water mass flux up to 0.3 µL mm^−2^ s^−1^. The slope in the linear region is 0.397 K/(µL mm^−2^ s^−1^), which provides a typical measure of the sensitivity for a calorimetric flow sensor. The maximum temperature rise of the heater was 4.69 K at zero flow. The ability of the designed single-probe sap flow sensor to measure reverse flow is demonstrated through the symmetrical response of the temperature difference of T_d _– T_u_ at backward flow, represented as a negative value of mass flux. Figure 3c, which compares the correlation between the ratio R and the mass flux attained through simulation and experimental calibration, indicates that the simulation results agree well with the experimental data, particularly at a low mass flux (maximum 3.4 % error at 3 µL mm^−2^ s^−1^). The standard deviation error of the ratio for each mass flux is on the order of 0.13 % compared with the full scale of 0.08, which is comparable to the resolution of the custom measurement circuit used in the calibration. Figure 3d displays the time response of the calorimetric sap flow sensor in response to heating; the ratio R was estimated every second for a total of 20 s. For a high mass flux (> 1 µL mm^−2^ s^−1^), the reading saturated after 10 s or less, while time response of the sensor increased with a decrease in water mass flux. For instance, at low mass flux (< 0.3 µL mm^−2^ s^−1^) that corresponds to the actual range of the sap flux density, the ratio R was not in thermal equilibrium even after 20 s of heating duration. This dependence of time response and mass flux can be explained by thermal time constant of the system, estimated as the product of thermal resistance and thermal capacitance, where thermal resistance of convective heat transfer increases with a decrease in water mass flux^44^. In our study, a 5 s heat duration was used as a compromise between high sensitivity and low power consumption. This short periodic heating with low power minimizes the heat stress that disturbs the physiological activities of plants such as growth, development, reproduction and yield^45^.

**Figure 4:**
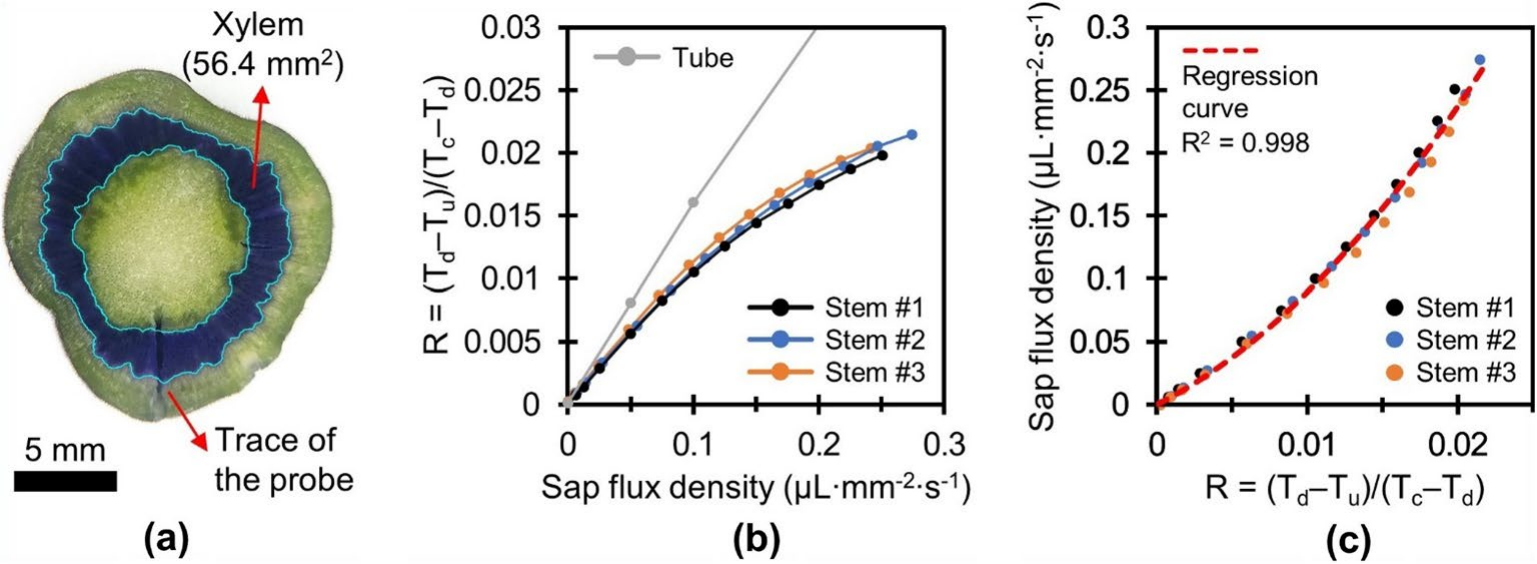
Stem calibration results and cross-sectional image of a tomato stem. (**a**) The stem perfused with 0.1 wt% crystal violet solution after calibration. Cyan lines indicate the boundary of the xylem. (**b**) Comparison of the calibration results for the tube and the stems. (**c**) Correlation between sensor response and SFD, fitted to a second-order polynomial model.

We obtained a calibration curve for three tomato stems using the setup, with the variation of connecting the excised tomato stem between the silicone tubes. The stem preparation process is described in the Methods section. Degassed and deionized water containing 20 mM potassium chloride (KCl) was used as the sap flowing through the stems. The SFD was

regulated up to 0.25 µL mm^−2^ s^−1^, a flux that is triple the maximum SFD observed during the field test on tomato trees. A cross-sectional image of the tomato stem used in the calibration is displayed in Fig. 4a. The stems were perfused with 0.1 wt% crystal violet solution to obtain the cross-sectional area of the xylem following calibration.

Figure 4b displays the calibration results that were obtained using the tube and three tomato stems. The sensitivities of the sensor assessed in the stem calibrations decrease to nearly 75% (0.299 K/(µL mm^−2^ s^−1^)) of that in the tube test when comparing the slopes at zero flow in the two cases. This reduction in sensitivity is mainly attributed to thermal diffusion through nonmoving xylem vessels and indirect contact between the sensor and moving sap. The standard deviation error of 10 consecutive measurements of the ratio R for each SFD was on the order of 0.5% compared with the full scale of 0.02, which is comparable noise level to that in the tube test; this implies that the sap flux in the stem is well regulated by the syringe pump without fluctuations that exceed the noise level of the custom readout electronics. Discrepancy in calibration results of stems was observed to be approximately 6 % at 0.2 µL mm^−2^ s^−1^. This uncertainty might result from anatomical and physiological heterogeneity of living entities (e.g., vessel diameter and distribution; radial and circumferential profile of sap flow). Figure 4c displays the correlation between the ratio R and the SFD fitted to a second-order polynomial model with an R^2^ value of 0.998 as follows:

2 $$\text{SFD}=292{R}^{2}+6.024R.$$

The standard deviation error of the estimated SFD at the midpoint (0.125 µL mm^−2^ s^−1^) of the calibration range, taking into account the noise level of the readout electronics, was 1.5 nL mm^−2^ s^−1^ and the maximum temperature rise of the heater at zero flow was 4.61 K.

No evidence of tylosis formation was observed in this study, even though the stem was collected one week after sensor insertion. We believe that tylosis formation was delayed or inhibited because the sensor was implanted without drilling that causes substantial mechanical and thermal stress near the drill hole^46^ and air inflow to the ruptured xylem vessels^47^.

### Field experiment

Sap flow measurements for tomato trees were conducted in a greenhouse under controlled environment, as described in the Methods section. Of the total four sensors, two were installed on different stems, and the other two were installed on the same stem at a vertical distance of approximately 5 cm on opposite sides. The sensor signals were transmitted to our data server every minute through Wi-Fi communication and converted into an SFD using equation (2).

**Figure 5:**
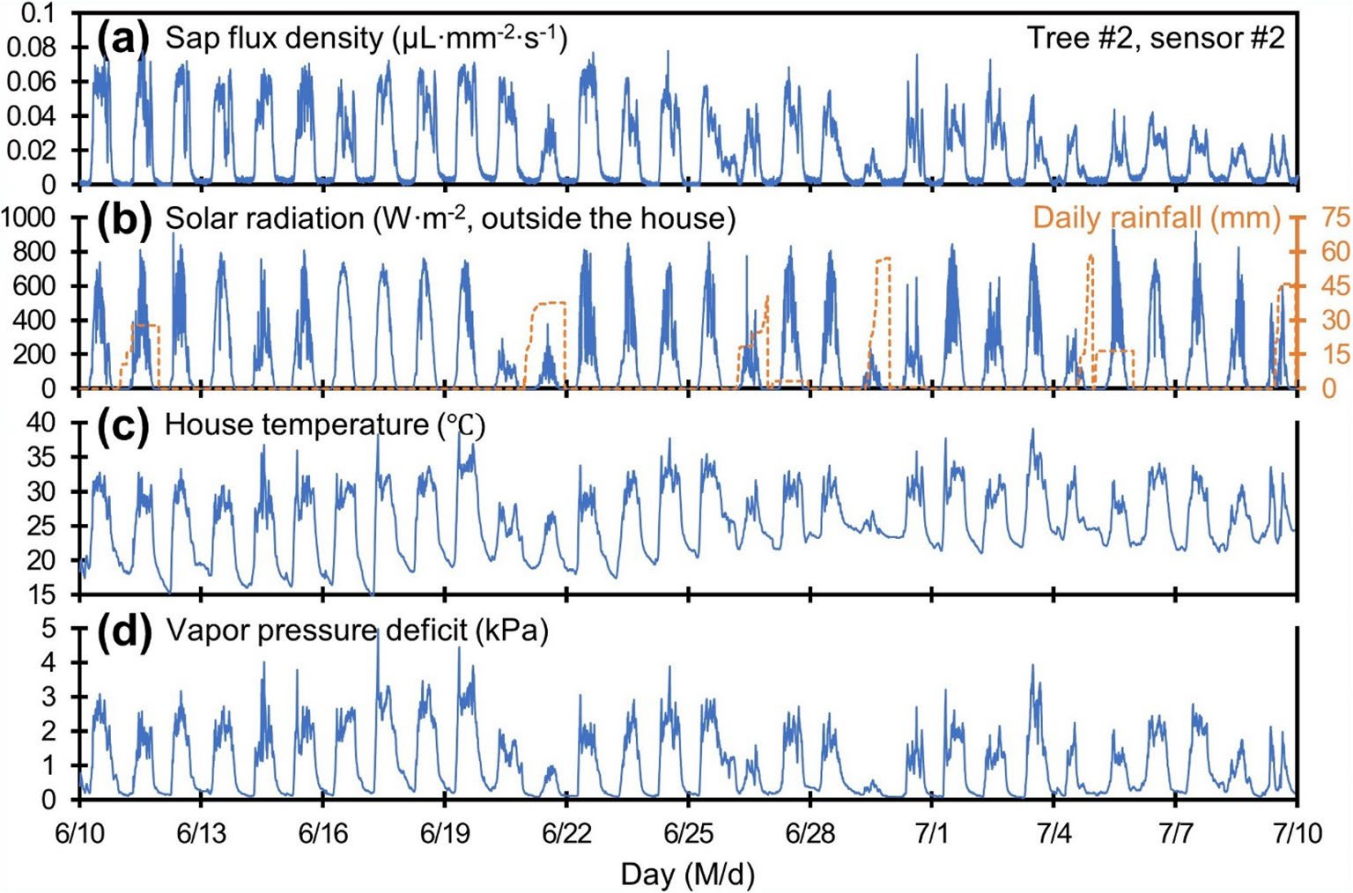
Field test result in the greenhouse over a one month period in relation to various environmental variables. (**a**) SFD of the tomato tree, (**b**) solar radiation and daily rainfall, (**c**) house temperature, and (**d**) vapor pressure deficit.

**Figure 6:**
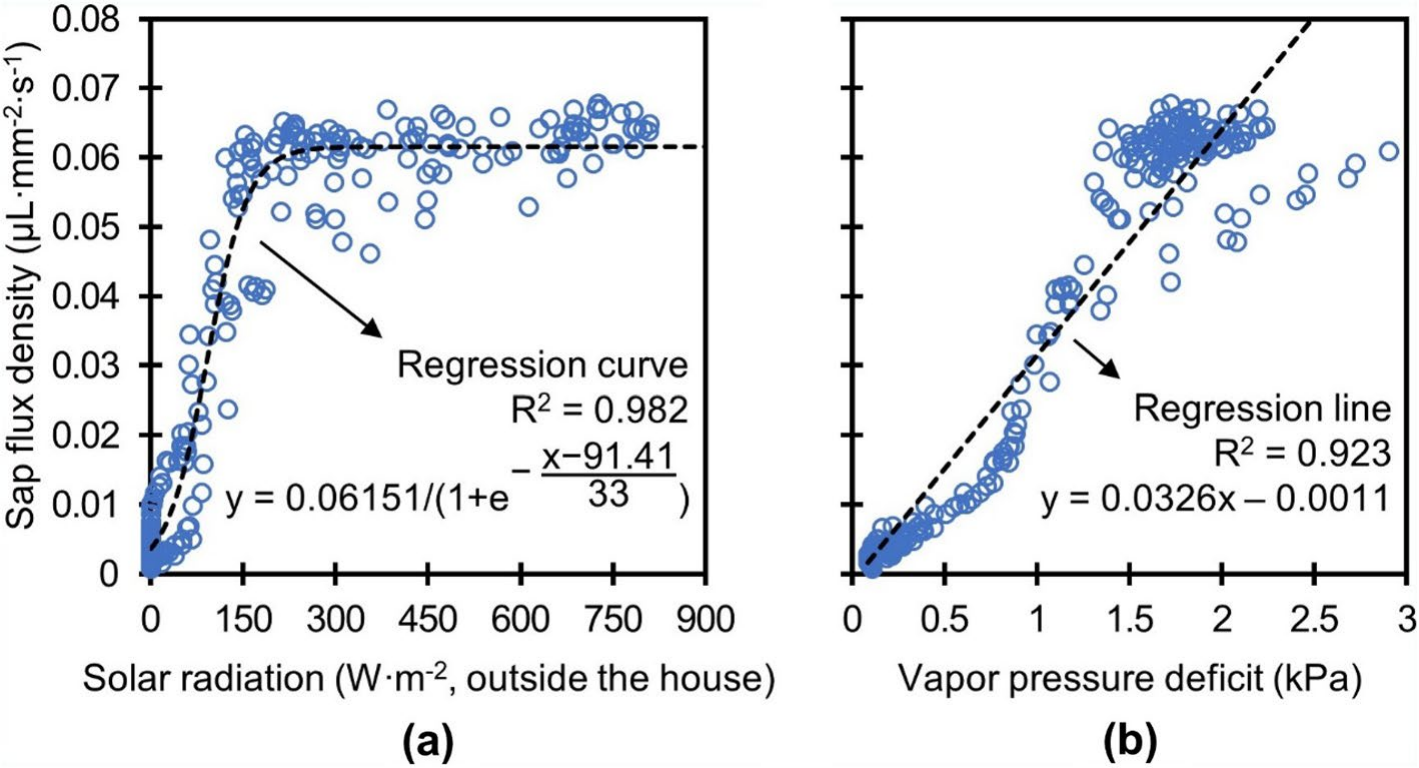
Relationship of SFD with (**a**) solar radiation and (**b**) vapor pressure deficit on 22nd of June.

Figure 5 displays the correlation of the SFD from one of the four sensors with various environmental variables measured over one month. The SFD was measured to be approximately 0.07 µL mm^−2^ s^−1^ during the daytime of the first week of field test, while it diminished after the rainy season began. The two main driving forces for transpiration, solar radiation and vapor pressure deficit (VPD), are compared to the SFD when sunshade is open throughout the day on 22nd of June in Fig. 6. The SFD increased proportionally with increments of the solar radiation until it reached a level of saturation (~ 0.065 µL mm^−2^ s^−1^) at higher solar radiation (Fig. 6a). This non-linear response of SFD may result from negative impact of high solar radiation on stomatal opening^48^ and is similar to the results (saturating at around 200 W m^−2^) observed in greenhouse tomato cultivation during rainy season in summer by Jo and Shin^49^. The SFD and VPD showed a linear relationship with an R^2^ of 0.92 (Fig. 6b).

**Figure 7:**
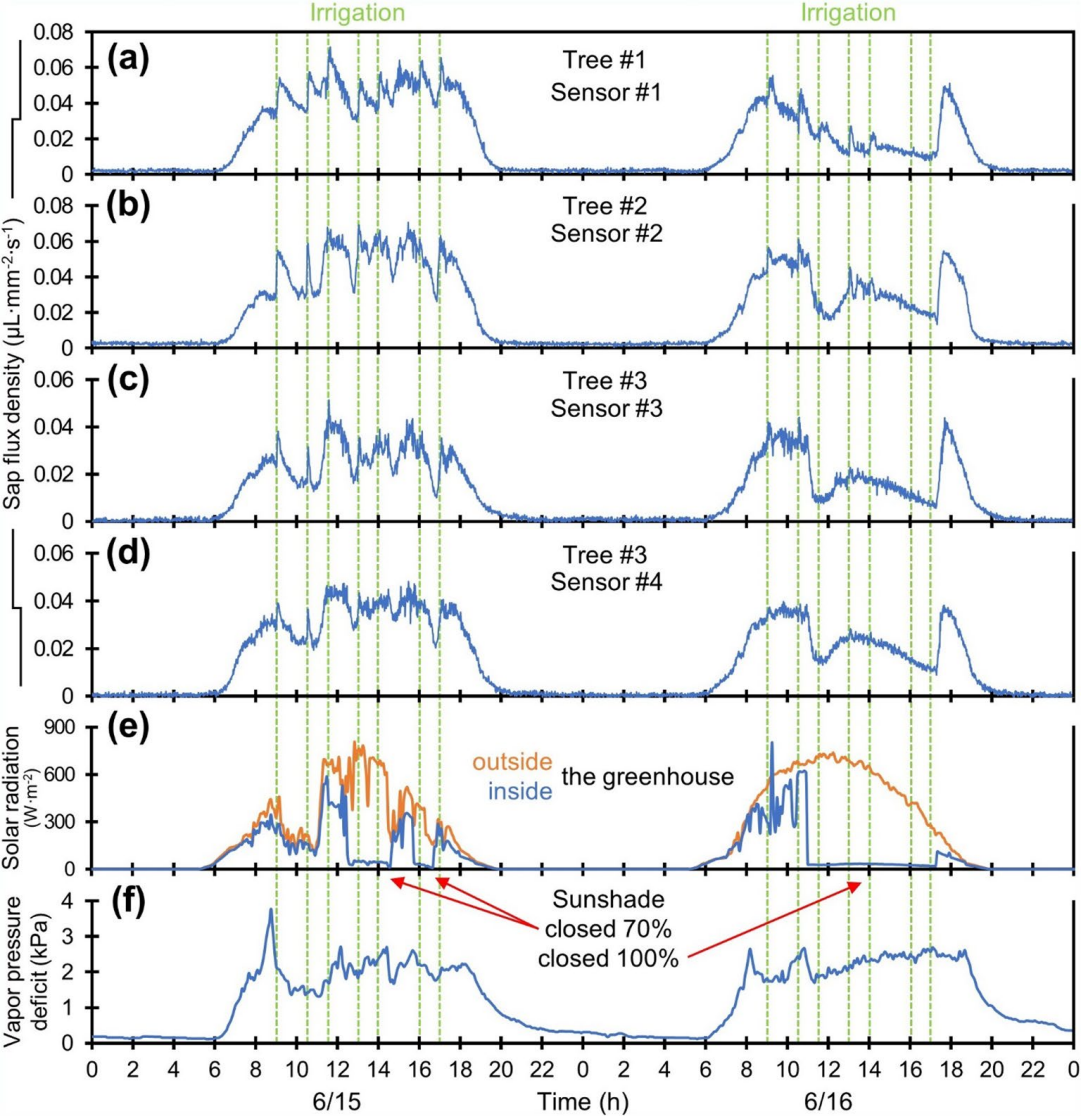
Enlarged field test results of four sensors for each (**a**) tree #1, (**b**) tree #2, and (**c**,**d**) tree #3 from 15th of June to 17th of June. (**e**) Solar radiation captured in and outside the greenhouse. (**f**) Vapor pressure deficit. Vertical green dotted lines mark the times irrigation begins at 9:00, 10:30, 11:30, 13:00, 14:00, 16:00 and 17:00.

Figure 7 displays the enlarged two-day measurement results of the four sensors on the three stems. The three trees showed comparable diurnal SFD patterns in response to the environmental variables. The tomato plant, which was quiescent during the night, showed a rapid increase in the SFD with sunrise as solar radiation and VPD increased. Through the day, the SFD remained in tune with these climatic conditions and fluctuated in accordance with the presence or absence of the sunshade; the SFD declined to a negligible extent at sunset, although the VPD still persisted at a weak level. The SFD captured by another sensor on the same stem exhibited a closer correlation in terms of magnitude and pattern than the results collected by a sensor on the other two stems (Fig. 7c,d). Interestingly, an upsurge in SFD was observed immediately after irrigation, which can be explained by abrupt changes in water potential near the roots. This upsurge was prominent during the first irrigation of the day and waned or disappeared as the irrigation was repeated, depending on the weather and the tree.

## Conclusion

We developed a micromachined calorimetric flow sensor that can be implanted into plant stems with minimal invasion to measure the SFD of plants. Complete isolation of the silicon substrates coupled with encapsulation of low-thermal-conductivity underfill epoxy minimized the thermal conduction among the sensing elements, thereby enhancing the sensitivity of the sensor. The newly designed calorimetric flow sensor is sufficiently robust for long-term field applications because the sensing component is structured without any brittle parts on the needle-like PCB. Moreover, the buried titanium resistive structure between the silicon substrate and PCB, realized with the flip-chip bonding technique, can avoid potential electrochemical corrosion, which was one of the causes of failure of the micromachined sap flow sensor^29^.

In the calibration test containing a tomato stem, the developed sensor exhibited a sensitivity of 0.299 K/(µL mm^−2^ s^−1^), which covers the full range of actual SFD of the tomato tree. The advantages of miniaturized calorimetric sap flow sensor, e.g., fast response, high sensitivity and low power consumption, facilitate sap flow measurement with a fairly low heating energy of 0.01 W for 5 s in one measurement cycle in comparison with conventional sap flow sensors (continuous 0.2 W in HD method^14^, continuous 0.06–0.08 W cm^−1^ in HFD method^16^, heat pulse of 9 W for 3 s in HR method^20^, periodic 0.067 W for 30 s in MEMS-based modified HD method^29^). This characteristic could be crucial for implementation in locations where access to the power grid is challenging. Three-element, or calorimetric, configuration of the developed sensor guarantees flow direction detection and consistent readings regardless of ambient temperature fluctuations.

Our month-long field test demonstrated an immediate shift in the SFD of tomato trees in response to sunshade and irrigation events, as well as a strong correlation between SFD and climatic variables. The micromachined calorimetric sap flow sensor, owing to its minimally invasive, robust, and low-power features, opens the door to new possibilities for sap flow monitoring.

## Methods

### Stem sample preparation process

Total three sap flow sensors were implanted into three different tomato trees in a greenhouse a week before cutting it to incorporate the wound-effect (e.g., tylosis formation) in the calibration curve. This pre-installation could reduce the potential bias caused by the wound. Before inserting the probe into the stem, the epidermis was carefully peeled off to remove hair-like trichomes, and an adequate amount of wound sealant (Lac balsam, Frunol Delicia) was applied to the stem to obstruct air inflow during insertion. The target penetration was 3.5 mm from the epidermis of the stem to the central line of the sensing elements. We excised the trees four hours prior to sunrise, when the sap flow was minimal to prevent cavitation of the xylem vessels. A segment 20 cm in length was cut from each tree and immediately stored in a plastic box full of water. Right before calibration, the stems were segmented again into a branchless section approximately 5 cm long and connected to a silicone tube using tube fittings and hose clamps.

### Method of field test

Sap flow measurements for tomato trees were conducted in a polyethylene greenhouse at the Urban Agricultural Research Center of Gwanak, Gwanak-gu, Seoul, South Korea, from June 10 to July 9, 2023. A total of 128 seedlings of 60-day-old tomato (‘Minichal’, Nongwoo Bio) were transplanted onto 32 rockwool slabs (20 × 100 × 8 cm) in two rows with a within-row spacing of 25 cm and a between-row spacing of 135 cm on February 10, 2023. During the measurement period, a blend of nutritive components (121.5 mL Floramiro, 121.5 mL Floragro and 174 mL Florabloom per 100 L of tap water, General Hydroponics) was supplied by means of a drip irrigation system with a volume of 150 mL for three minutes per irrigation. The number of irrigations and their timing were regulated according to the climatic conditions; for instance, on a sunny day, the solution was dispensed seven times at 9:00, 10:30, 11:30, 13:00, 14:00, 16:00, and 17:00. An air conditioner in combination with a horizontal sunshade on the greenhouse ceiling was deployed to maintain the temperature within the greenhouse at a maximum of 35 °C. Solar radiation and daily rainfall were recorded at a weather station (WS-2902, Ambient Weather), which was installed in an open field adjacent to the greenhouse, while the weather station located on the greenhouse ceiling logged the internal temperature, humidity, and solar radiation at five-minute intervals. One week prior to measurement, four sensors were implanted on the stems of the tomato trees at approximately 70 cm from the root, with a target depth of 3.5 mm.

### Ethical declarations

This study was complied with the relevant institutional, national, and international guidelines and legislations. The permission was obtained for experimental and field test on tomato plants including the collection of plant material from the Urban Agricultural Research Center of Gwanak.

## Data Availability

The data supporting this study are available upon a reasonable request to the corresponding author.

## References

[1] Peixoto Neto AML (2022). Linking evapotranspiration seasonal cycles to the water balance of headwater catchments with contrasting land uses. Hydrol. Process..

[2] Naithani KJ, Ewers BE, Pendall E (2012). Sap flux-scaled transpiration and stomatal conductance response to soil and atmospheric drought in a semi-arid sagebrush ecosystem. J. Hydrol..

[3] Wang H (2012). Ozone uptake by adult urban trees based on sap flow measurement. Environ. Pollut..

[4] Pataki DE, McCarthy HR, Litvak E, Pincetl S (2011). Transpiration of urban forests in the Los Angeles metropolitan area. Ecol. Appl..

[5] Doronila AI, Forster MA (2015). Performance measurement via sap flow monitoring of three eucalyptus species for mine site and dryland salinity phytoremediation. Int. J. Phytoremediation.

[6] Ferreira MI, Silvestre J, Conceição N, Malheiro AC (2012). Crop and stress coefficients in rainfed and deficit irrigation vineyards using sap flow techniques. Irrig. Sci..

[7] Saitta D (2021). Adaptation of citrus orchards to deficit irrigation strategies. Agric. Water Manag..

[8] Fernández JE, Green SR, Caspari HW, Diaz-Espejo A, Cuevas MV (2007). The use of sap flow measurements for scheduling irrigation in olive, apple and Asian pear trees and in grapevines. Plant Soil.

[9] Yoon BH (2020). Analysis of plant and fruit growth and yield under a micro sap flow sensor automated irrigation in substrate hydroponics for tomato cultivation. Acta Hortic..

[10] McElrone AJ, Grant JA, Kluepfel DA (2010). The role of tyloses in crown hydraulic failure of mature walnut trees afflicted by apoplexy disorder. Tree Physiol..

[11] Urban J, Dvořák M (2014). Sap flow-based quantitative indication of progression of Dutch elm disease after inoculation with Ophiostoma novo-ulmi. Trees.

[12] Ouadi L (2019). Ecophysiological impacts of Esca, a devastating grapevine trunk disease, on Vitis vinifera L. PLoS One.

[13] Huber B (1932). Beobachtung und messung pflanzicher sartströme. Ber. Dtsch. Bot. Ges..

[14] Granier A (1985). Une nouvelle méthode pour la mesure du flux de sève brute dans le tronc des arbres. Ann. For. Sci..

[15] King LV (1914). On the convection of heat from small cylinders in a stream of fluid : Determination of the convection constants of small platinum wires, with applications to hot-wire anemometry. Proc. R. Soc. Lond. A.

[16] Nadezhdina N, Vandegehuchte MW, Steppe K (2012). Sap flux density measurements based on the heat field deformation method. Trees.

[17] Marshall DC (1958). Measurement of sap flow in conifers by heat transport. Plant Physiol..

[18] Swanson RH, Whitfield WA (1981). A numerical analysis of heat pulse velocity theory and practice. J. Exp. Bot..

[19] Cohen Y, Fuchs M, Green GC (1981). Improvement of the heat pulse method for determining sap flow in trees. Plant Cell Environ..

[20] Burgess SSO (2001). An improved heat pulse method to measure low and reverse rates of sap flow in woody plants. Tree Physiol..

[21] Vandegehuchte MW, Steppe K (2013). Sap-flux density measurement methods: working principles and applicability. Funct. Plant Biol..

[22] Marañón-Jiménez S (2018). X-ray computed microtomography characterizes the wound effect that causes sap flow underestimation by thermal dissipation sensors. Tree Physiol..

[23] Green S, Clothier B, Jardine B (2003). Theory and practical application of heat pulse to measure sap flow. Agron. J..

[24] Neely D (1988). Wound closure rates on trees. Arboric. Urban For..

[25] Dujesiefken D, Liese W, Shortle W, Minocha R (2005). Response of beech and oaks to wounds made at different times of the year. Eur. J. For. Res..

[26] Sun Q, Rost TL, Matthews MA (2006). Pruning-induced tylose development in stems of current-year shoots of Vitis vinifera (Vitaceae). Am. J. Bot..

[27] Sun Q, Rost TL, Matthews MA (2008). Wound-induced vascular occlusions in Vitis vinifera (Vitaceae): Tyloses in summer and gels in winter. Am. J. Bot..

[28] Ren R, von der Crone J, Horton R, Liu G, Steppe K (2020). An improved single probe method for sap flow measurements using finite heating duration. Agric. For. Meteorol..

[29] Baek S, Jeon E, Park KS, Yeo K-H, Lee J (2018). Monitoring of water transportation in plant stem with microneedle sap flow sensor. J. Microelectromech. Syst..

[30] Hara Y (2018). Microscale xylem sap flow sensor facilitating the smultaneous measurement of flow velocity and direction. Proceedings.

[31] Yano Y (2018). Phloem-sap-dynamics sensor device for monitoring photosynthates transportation in plant shoots. Jpn. J. Appl. Phys..

[32] Rabbel I, Diekkrüger B, Voigt H, Neuwirth B (2016). Comparing ∆Tmax determination approaches for granier-based sapflow estimations. Sensors.

[33] Ejeian F (2019). Design and applications of MEMS flow sensors: A review. Sens. Actuator A Phys..

[34] Lammerink TSJ, Tas NR, Elwenspoek M, Fluitman JHJ (1993). Micro-liquid flow sensor. Sens. Actuator A Phys..

[35] Nguyen NT, Dötzel W (1997). Asymmetrical locations of heaters and sensors relative to each other using heater arrays: A novel method for designing multi-range electrocaloric mass-flow sensors. Sens. Actuator A Phys..

[36] Vilares R (2010). Fabrication and testing of a SU-8 thermal flow sensor. Sens. Actuators B Chem..

[37] Shimohira C (2020). Development of micromachined flow sensor for drip infusion system. Microsyst. Technol..

[38] Ren R (2017). The effects of probe misalignment on sap flux density measurements and in situ probe spacing correction methods. Agric. For. Meteorol..

[39] Elwenspoek M, Wiegerink R (2012). Mechanical Microsensors.

[40] Tas NR (2000). Toward thermal flow-sensing with pL/s resolution. Proc. SPIE.

[41] Xu W (2023). A sub-5mW monolithic CMOS-MEMS thermal flow sensing SoC with ±6 m/s linear range. IEEE J. Solid-State Circuits.

[42] Almerico JP, Werbaneth PF, Cho YK (2002). Feasibility analysis for dry plasma scribe lane etch for die separation in compound semiconductors. III-Vs Rev..

[43] Ayers JE, Kujofsa T, Rago P, Raphael J (2016). Heteroepitaxy of Semiconductors: Theory, Growth, and Characterization.

[44] Sosna C, Walter T, Lang W (2011). Response time of thermal flow sensors with air as fluid. Sens. Actuator A Phys..

[45] Hasanuzzaman M, Nahar K, Alam MM, Roychowdhury R, Fujita M (2013). Physiological, biochemical, and molecular mechanisms of heat stress tolerance in plants. Int. J. Mol. Sci..

[46] Barrett DJ, Hatton TJ, Ash JE, Ball MC (1995). Evaluation of the heat pulse velocity technique for measurement of sap flow in rainforest and eucalypt forest species of south-eastern Australia. Plant Cell Environ..

[47] Barnett JR, Burley J (2004). Xylem physiology. Encyclopedia of Forest Sciences.

[48] O’Carrigan A (2014). Effects of light irradiance on stomatal regulation and growth of tomato. Environ. Exp. Bot..

[49] Jo WJ, Shin JH (2021). Development of a transpiration model for precise tomato (*Solanum lycopersicum* L.) irrigation control under various environmental conditions in greenhouse. Plant Physiol. Biochem..

